# Supplementary material for: Hole migration in cytochrome P450
Source: QRB Discov. 2026 May 4;7:e5. doi: 10.1017/qrd.2026.10021 (PMC13161928; doi:10.1017/qrd.2026.10021)
Supplement: Gray and Winkler supplementary material [file S2633289226100210sup001.pdf]

Supplementary Material

Hole Migration in Cytochrome P450

Harry B. Gray*[1] and Jay R. Winkler*[1]

[1] Beckman Institute

California Institute of Technology

Pasadena, CA 91125

USA

* hbgray@caltech.edu

* winklerj@caltech.edu

Contents

Page

Kinetics Modeling: general 2

Kinetics Modeling: CYP158A2, Figures S1-S8 4

Kinetics Modeling: CYP119, Figures S9-S16 6

Kinetics Modeling: CYP102A1, Figures S17-S34 9

Kinetics Modeling: CYP3A4, Figures S35-S48 13

Kinetics Modeling: general

The kinetics of heme hole survival for a protein with *n* redox active residues and one heme are described by a system of (n+1) coupled ordinary first-order differential equations (Eq. 1).

$$\frac{d\mathbf{u}}{dt} = \mathbf{A}\mathbf{u} \tag{1}$$

The vector $\mathbf{u}$ represents the concentrations of the (n+1) components of the model and the (n+1)×(n+1) matrix $\mathbf{A}$ is composed of all rate constants describing electron flow between all pairs of redox-active groups. The diagonal elements of $\mathbf{A}$ are given by the negative of the sum of all rate constants for hole transfer out of the redox-active residues. The off-diagonal elements of $\mathbf{A}$ correspond to rate constants for hole transfer into the redox-active residues. The elements of matrix $\mathbf{A}$ are defined using Eq. (2) with parameters defined in the main text.

$$k_{\mathrm{ET}}(\Delta G^{\mathrm{o}}, \lambda, \beta, r) = 10^{13} e^{-\beta(r-r_{\mathrm{o}})} e^{\left\{-\frac{(\Delta G^{\mathrm{o}}+\lambda)^2}{4\lambda k_{\mathrm{B}}T}\right\}} \tag{2}$$

The eigenvalue problem described by eq. 1 was solved by numerically using the MATLAB (Mathworks, Inc.) function *eig* to obtain eigenvalues (ε_1, ε_2, … ε_{n+1}) and a matrix of eigenvectors $\mathbf{S}$. Time dependent concentrations for all components are given by eq. 3. The initial value vector $\mathbf{u_0}$

$$\mathbf{u}(t) = \mathbf{S}\begin{bmatrix} e^{\varepsilon_1 t} & 0 & 0 \\ 0 & \ddots & 0 \\ 0 & 0 & e^{\varepsilon_{n+1} t} \end{bmatrix}\mathbf{S}^{-1}\mathbf{u_0} \tag{3}$$

prescribes the hole population on the heme to be equal to 1 at t = 0 and 0 for all other residues.

Each residue is assigned a nominal formal potential on the basis of their hydrogen bonding and surface exposure as defined in Table 1 of the main manuscript. Time-dependent hole populations are calculated as described above. The MATLAB *randn* random number generating function was used to create a distribution of 10,000 formal potentials for each residue centered at the nominal value with a standard deviation of 0.1 V. Time dependent hole populations were calculated for the 10,000

combinations of potentials. Statistics of hole survival times, inflows, and outflows were determined from the 10,001 sets of time-dependent hole populations.

Kinetics Modeling: CYP158A2

The nominal potentials of redox-active residues in the CYP158A2 structure (PDB ID: 1S1F) are given in the following table. In flow plots, symbols preceding the residue name correspond to their surface exposure: buried, no symbol; buried and H-bonded to H_2O, #; surface exposed, *.

Residue	Nominal E° (vs. NHE)
Heme	1.0
Tyr198	0.90
Tyr315	0.95
Tyr318	0.95
Tyr352	0.90
Trp16	1.05
Trp50	1.0
Trp52	1.1
Trp174	1.0
Trp284	1.05
Trp404	1.05
Cys158	1.0

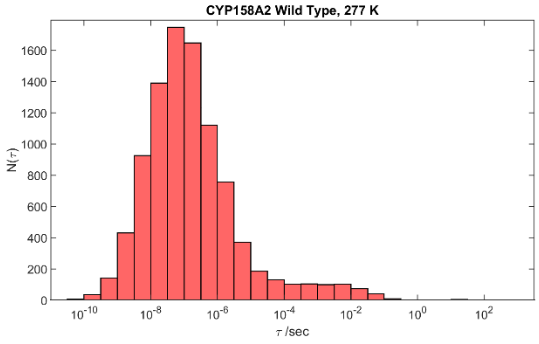

Figure S1. Distributions of calculated CI lifetimes (τ) for wild type CYP158A2 at 277 K. Mean value is 170 ns.

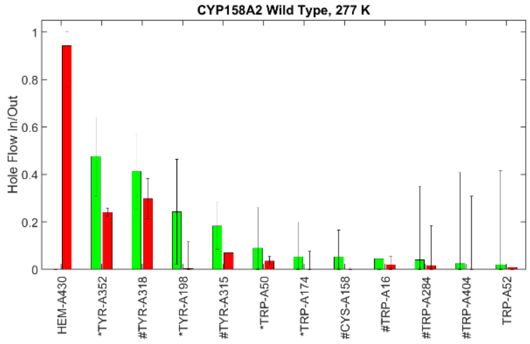

Figure S2. Calculated hole flow for redox active residues in CYP158A2. Green bars correspond to holes flowing into the residue and red bars correspond to holes flowing out. Error bars correspond to one standard deviation.

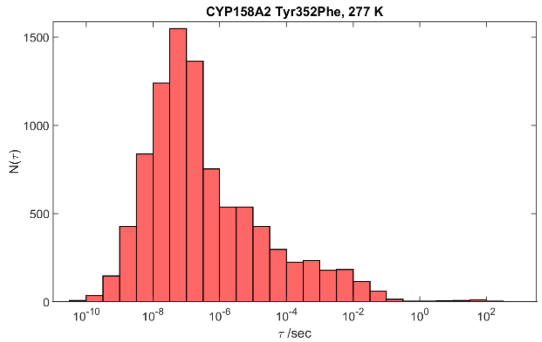

Figure S3. Distributions of calculated CI lifetimes (τ) for CYP158A2 Tyr352Phe mutant at 277 K. Mean value is 378 ns.

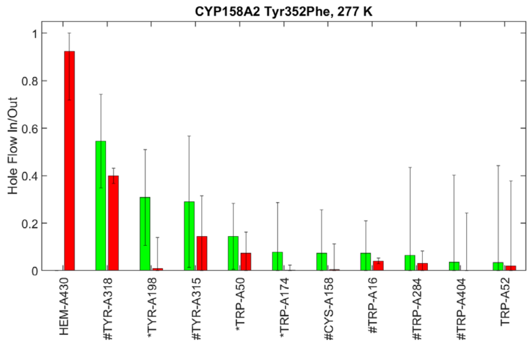

Figure S4. Calculated hole flow for redox active residues in CYP158A2 Tyr352Phe mutant. Green bars correspond to holes flowing into the residue and red bars correspond to holes flowing out. Error bars correspond to one standard deviation.

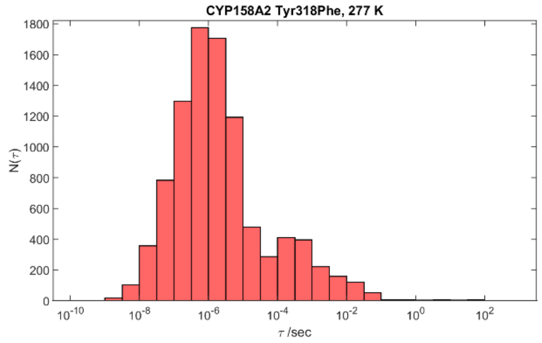

Figure S5. Distributions of calculated CI lifetimes (τ) for CYP158A2 Tyr318Phe mutant at 277 K. Mean value is 2.3 μs.

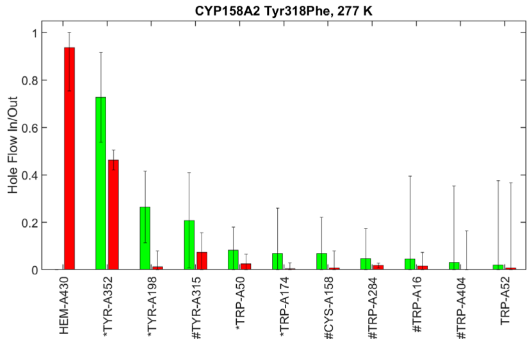

Figure S6. Calculated hole flow for redox active residues in CYP158A2 Tyr318Phe mutant. Green bars correspond to holes flowing into the residue and red bars correspond to holes flowing out. Error bars correspond to one standard deviation.

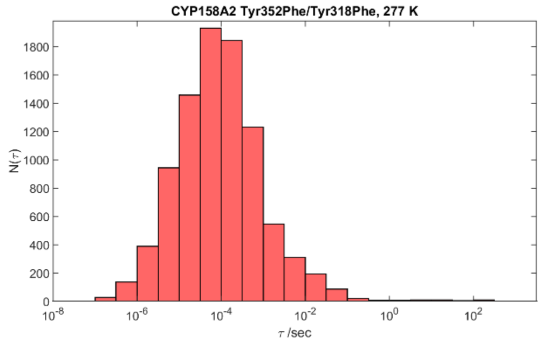

Figure S7. Distributions of calculated CI lifetimes (τ) for CYP158A2 Tyr352Phe/Tyr318Phe double mutant at 277 K. Mean value is 94 μs.

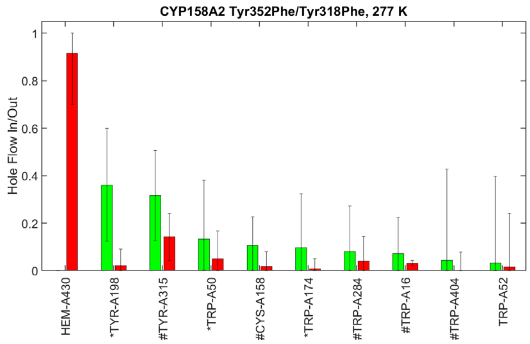

Figure S8. Calculated hole flow for redox active residues in CYP158A2 Tyr352Phe/Tyr318Phe double mutant. Green bars correspond to holes flowing into the residue and red bars correspond to holes flowing out. Error bars correspond to one standard deviation.

Kinetics Modeling: CYP119

The nominal potentials of redox-active residues in Chain A of the CYP119 structure (PDB ID: 1IO7) are given in the following table. In flow plots, symbols preceding the residue name correspond to their surface exposure: buried, no symbol; buried and H-bonded to H_2O, #; surface exposed, *.

Residue	Nominal E° (vs. NHE)
Heme	1.0
Tyr2	0.90
Tyr15	0.90
Tyr16	0.90
Tyr26	0.95
Tyr28	0.90
Tyr46	0.95
Tyr66	0.95
Tyr168	0.90
Tyr174	0.95
Tyr250	0.95
Tyr277	0.90
Tyr357	0.90
Trp4	1.0
Trp21	1.1
Trp147	1.1
Trp231	1.05
Trp281	1.05

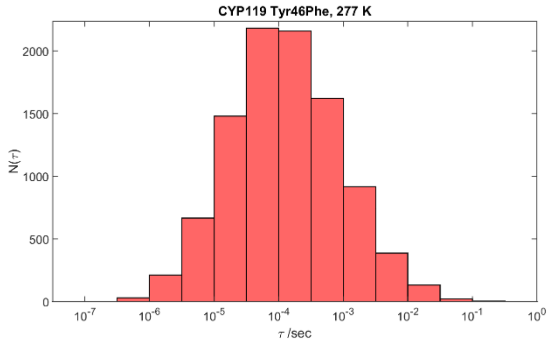

Figure S9. Distributions of calculated CI lifetimes (τ) for CYP119 Tyr46Phe mutant at 277 K. Mean value is 126 μs.

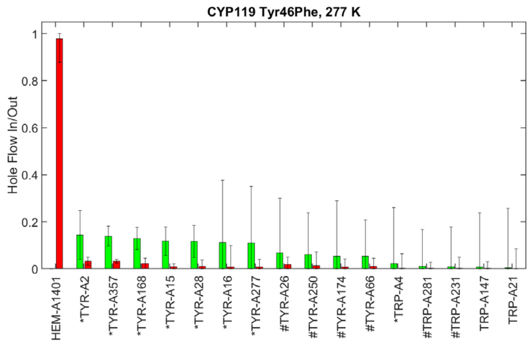

Figure S10. Calculated hole flow for redox active residues in CYP119 Tyr46Phe mutant. Green bars correspond to holes flowing into the residue and red bars correspond to holes flowing out. Error bars correspond to one standard deviation.

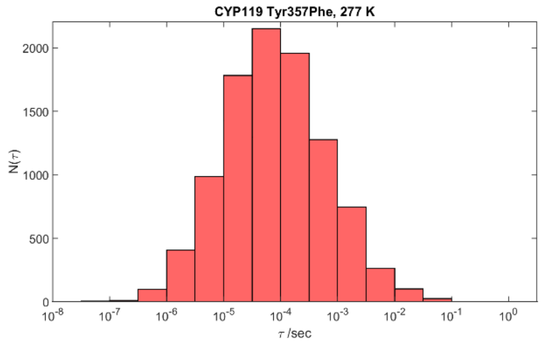

Figure S11. Distributions of calculated CI lifetimes (τ) for CYP119 Tyr357Phe mutant at 277 K. Mean value is 81 ns.

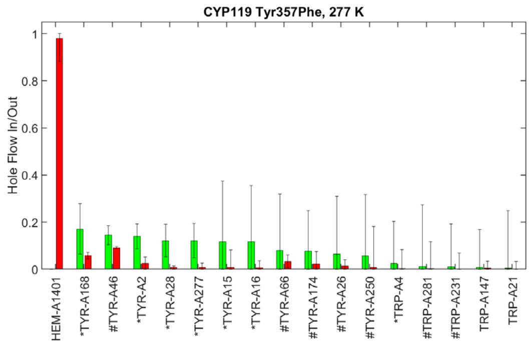

Figure S12. Calculated hole flow for redox active residues in CYP119 Tyr357Phe mutant. Green bars correspond to holes flowing into the residue and red bars correspond to holes flowing out. Error bars correspond to one standard deviation.

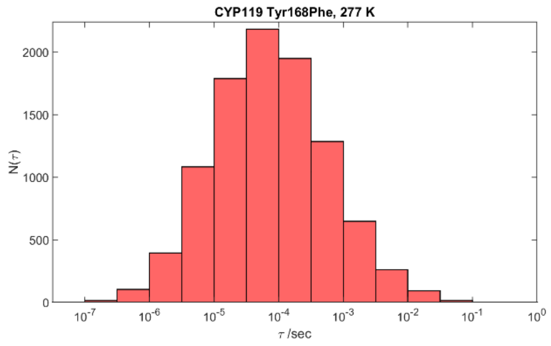

Figure S13. Distributions of calculated CI lifetimes (τ) for CYP119 Tyr168Phe mutant at 277 K. Mean value is 75 μs.

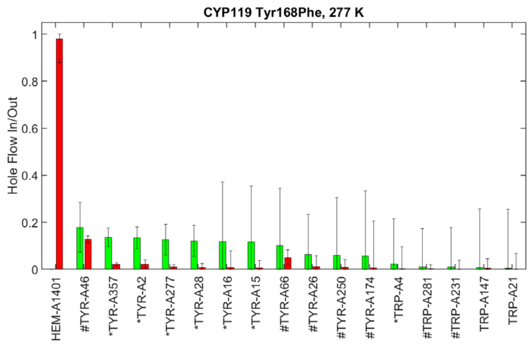

Figure S14. Calculated hole flow for redox active residues in CYP119 Tyr168Phe mutant. Green bars correspond to holes flowing into the residue and red bars correspond to holes flowing out. Error bars correspond to one standard deviation.

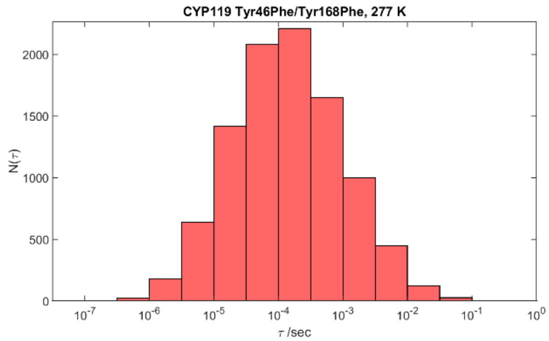

Figure S15. Distributions of calculated CI lifetimes (τ) for CYP119 Tyr46Phe/Tyr168Phe double mutant at 277 K. Mean value is 140 μs.

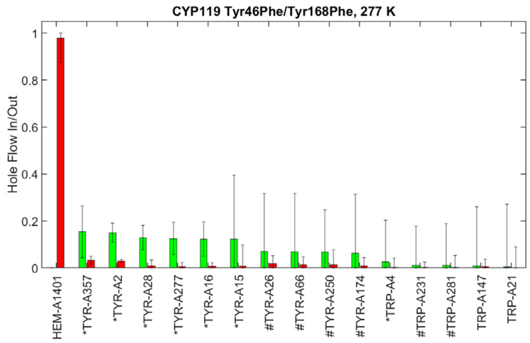

Figure S16. Calculated hole flow for redox active residues in CYP119 Tyr46Phe/Tyr168Phe double mutant. Green bars correspond to holes flowing into the residue and red bars correspond to holes flowing out. Error bars correspond to one standard deviation.

Kinetics Modeling: CYP102A1

The nominal potentials of redox-active residues in Chain A of the CYP102A1 structure (PDB ID: 2IJ2) are given in the following table. In flow plots, symbols preceding the residue name correspond to their surface exposure: buried, no symbol; buried and H-bonded to H_2O, #; surface exposed, *.

Residue	Nominal E° (vs. NHE)
Heme	1.0
Tyr51	0.95
Tyr115	0.95
Tyr160	0.90
Tyr166	0.90
Tyr198	0.90
Tyr256	0.90
Tyr278	0.95
Tyr305	0.95
Tyr313	0.95
Tyr334	0.90
Tyr345	0.95
Tyr429	0.95
Trp90	1.0
Trp96	1.05
Trp130	1.05
Trp325	1.05
Trp367	1.05
Cys62	1.05
Cys156	1.0

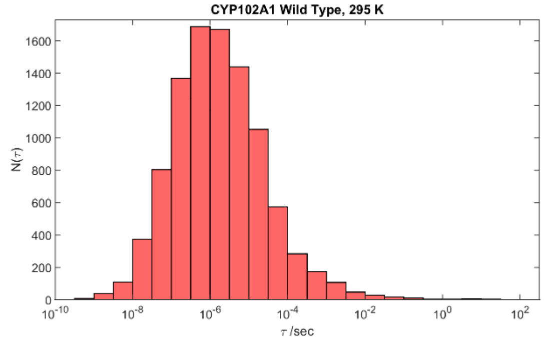

Figure S17. Distributions of calculated CI lifetimes (τ) for wild-type CYP102A1 at 295 K. Mean value is 1.7 μs.

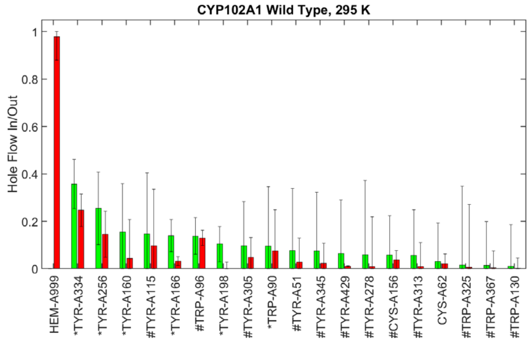

Figure S18. Calculated hole flow for redox active residues in wild-type CYP102A1. Green bars correspond to holes flowing into the residue and red bars correspond to holes flowing out. Error bars correspond to one standard deviation.

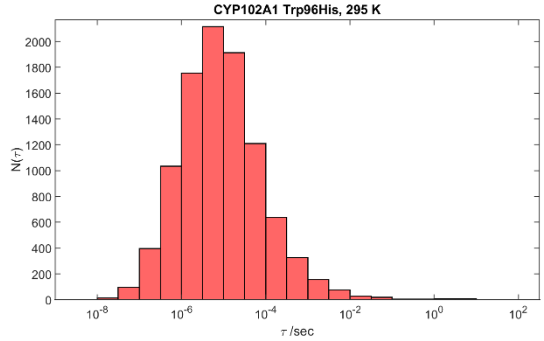

Figure S19. Distributions of calculated CI lifetimes (τ) for CYP102A1 Trp96His mutant at 295 K. Mean value is 9.0 μs.

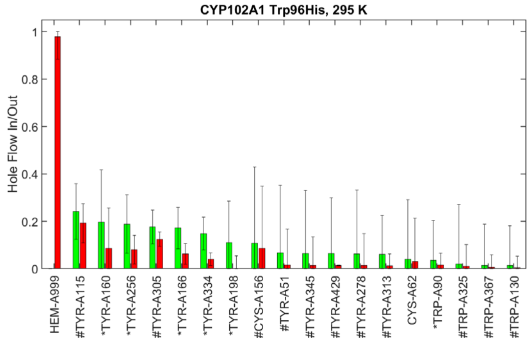

Figure S20. Calculated hole flow for redox active residues in CYP102A1 Trp96His mutant. Green bars correspond to holes flowing into the residue and red bars correspond to holes flowing out. Error bars correspond to one standard deviation.

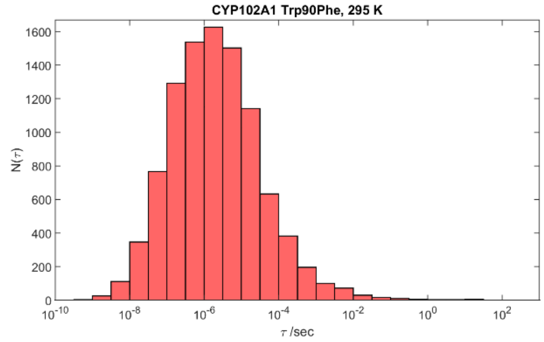

Figure S21. Distributions of calculated CI lifetimes (τ) for CYP102A1 Trp90Phe mutant at 295 K. Mean value is 2.1 μs.

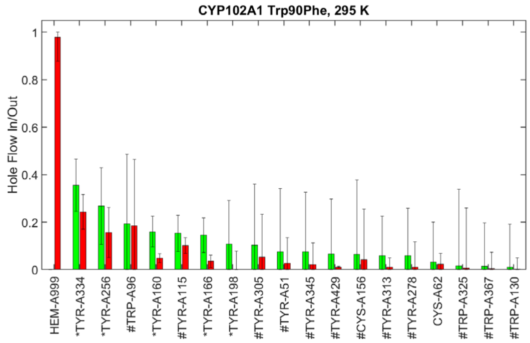

Figure S22. Calculated hole flow for redox active residues in CYP102A1 Trp90Phe mutant. Green bars correspond to holes flowing into the residue and red bars correspond to holes flowing out. Error bars correspond to one standard deviation.

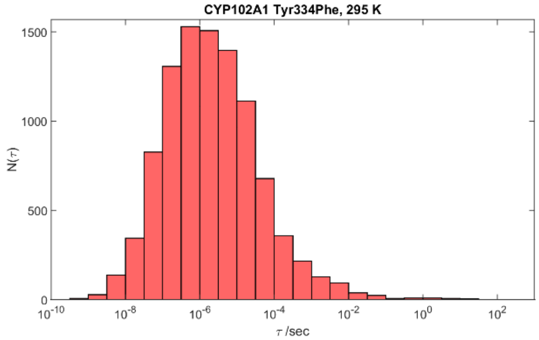

Figure S23. Distributions of calculated CI lifetimes (τ) for CYP102A1 Tyr334Phe mutant at 295 K. Mean value is 2.2 μs.

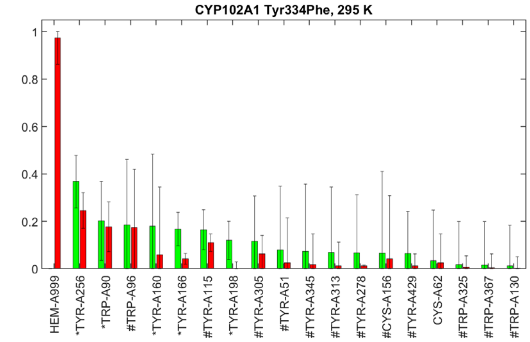

Figure S24. Calculated hole flow for redox active residues in CYP102A1 Tyr334Phe mutant. Green bars correspond to holes flowing into the residue and red bars correspond to holes flowing out. Error bars correspond to one standard deviation.

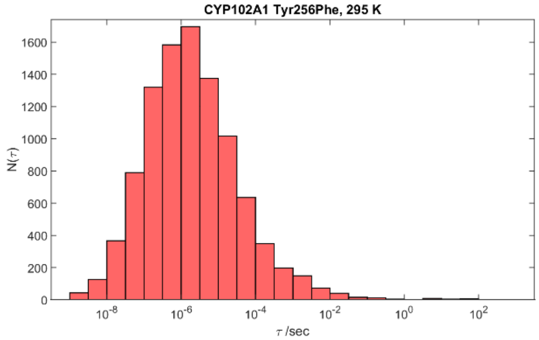

Figure S25. Distributions of calculated CI lifetimes (τ) for CYP102A1 Tyr256Phe mutant at 295 K. Mean value is 2.0 μs.

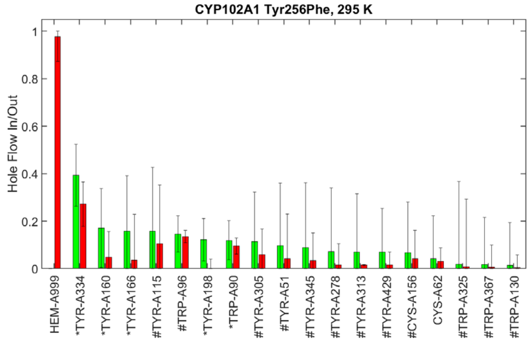

Figure S26. Calculated hole flow for redox active residues in CYP102A1 Tyr256Phe mutant. Green bars correspond to holes flowing into the residue and red bars correspond to holes flowing out. Error bars correspond to one standard deviation.

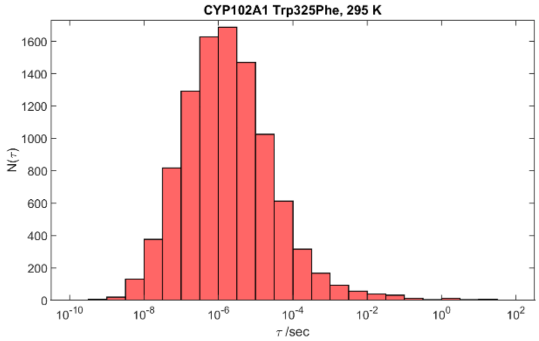

Figure S27. Distributions of calculated CI lifetimes (τ) for CYP102A1 Trp325Phe mutant at 295 K. Mean value is 1.9 μs.

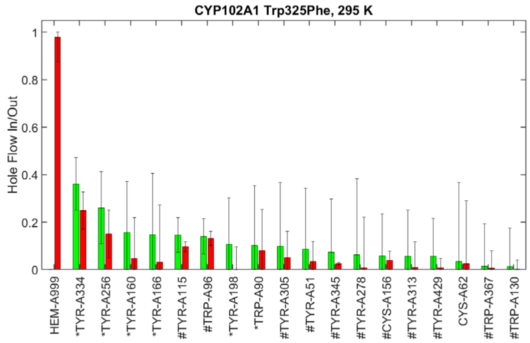

Figure S28. Calculated hole flow for redox active residues in CYP102A1 Trp325Phe mutant. Green bars correspond to holes flowing into the residue and red bars correspond to holes flowing out. Error bars correspond to one standard deviation.

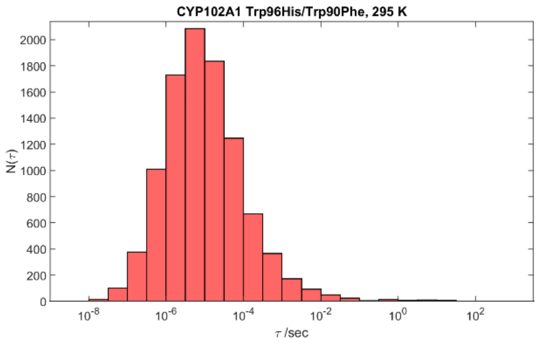

Figure S29. Distributions of calculated CI lifetimes (τ) for CYP102A1 Trp96His/Trp90Phe double mutant at 295 K. Mean value is 9.9 μs.

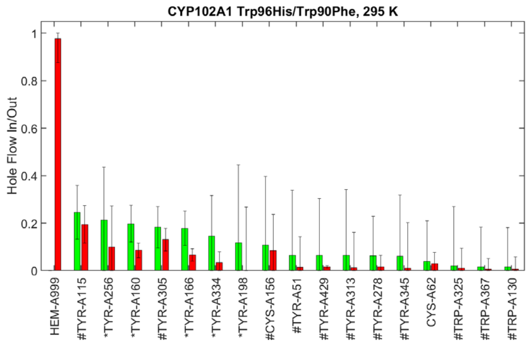

Figure S30. Calculated hole flow for redox active residues in CYP102A1 Trp96His/Trp90Phe double mutant. Green bars correspond to holes flowing into the residue and red bars correspond to holes flowing out. Error bars correspond to one standard deviation.

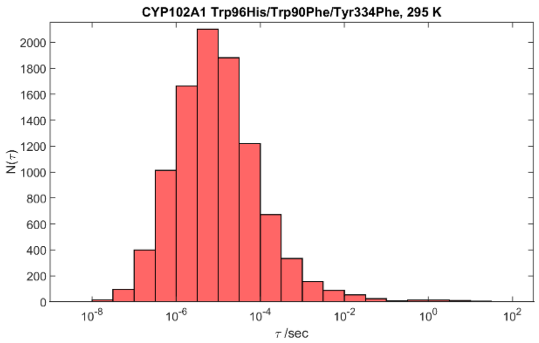

Figure S31. Distributions of calculated CI lifetimes (τ) for CYP102A1 Trp96His/Trp90Phe/Tyr334Phe triple mutant at 295 K. Mean value is 9.8 μs.

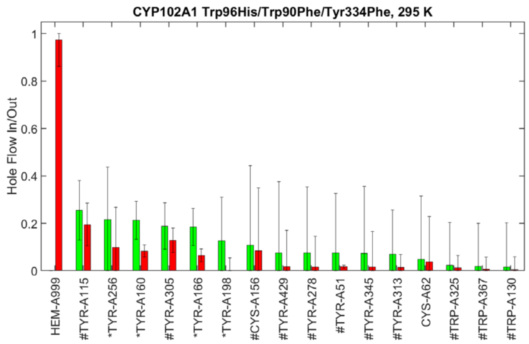

Figure S32. Calculated hole flow for redox active residues in CYP102A1 Trp96His/Trp90Phe/Tyr334Phe triple mutant. Green bars correspond to holes flowing into the residue and red bars correspond to holes flowing out. Error bars correspond to one standard deviation.

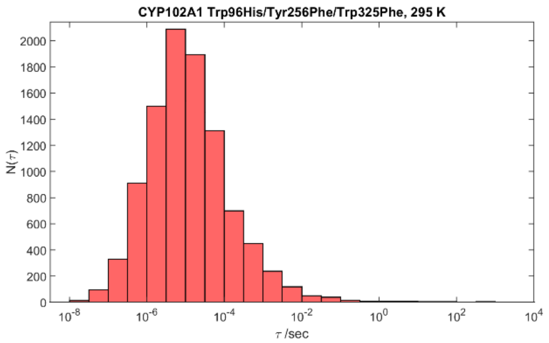

Figure S33. Distributions of calculated CI lifetimes (τ) for CYP102A1 Trp96His/Tyr256Phe/Trp325Phe triple mutant at 295 K. Mean value is 12.6 μs.

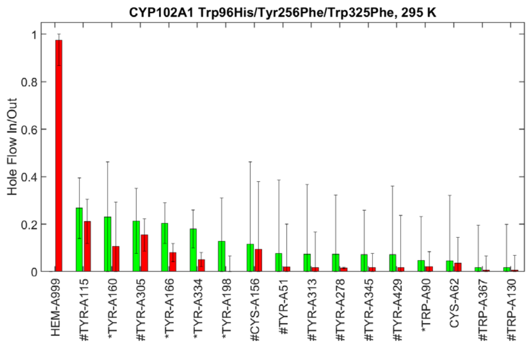

Figure S34. Calculated hole flow for redox active residues in CYP102A1 Trp96His/Tyr256Phe/Trp325Phe triple mutant. Green bars correspond to holes flowing into the residue and red bars correspond to holes flowing out. Error bars correspond to one standard deviation.

Kinetics Modeling: CYP3A4

The nominal potentials of redox-active residues in the CYP102A1 structure (PDB ID: 5VCC) are given in the following table. In flow plots, symbols preceding the residue name correspond to their surface exposure: buried, no symbol; buried and H-bonded to H_2O, #; surface exposed, *.

Residue	Nominal E° (vs. NHE)
Heme	1.0
Tyr53	0.95
Tyr68	0.90
Tyr75	0.95
Tyr99	0.90
Tyr152	0.95
Tyr179	0.95
Tyr307	0.95
Tyr347	0.95
Tyr355	0.95
Tyr399	0.95
Tyr407	0.90
Tyr430	0.90
Tyr432	0.95
Trp72	1.05
Trp126	1.1
Trp408	1.1
Cys58	1.0
Cys64	1.0
Cys98	1.0
Cys468	1.05

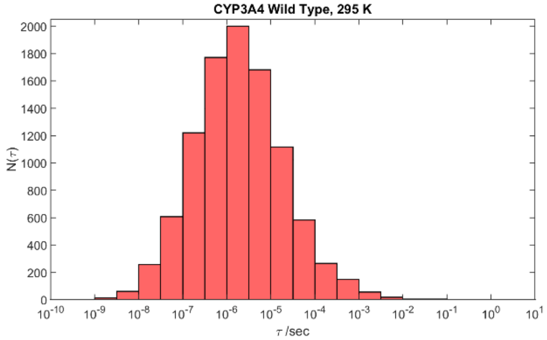

Figure S35. Distributions of calculated CI lifetimes (τ) for wild-type CYP3A4 at 295 K. Mean value is 1.9 μs.

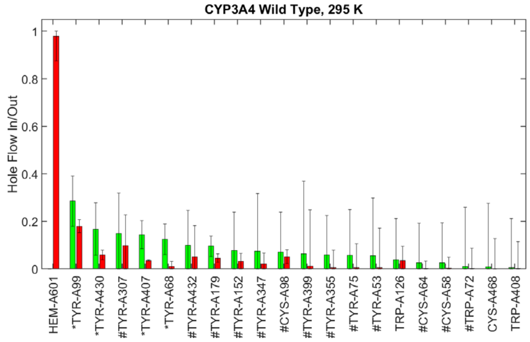

Figure S36. Calculated hole flow for redox active residues in wild-type CYP3A4. Green bars correspond to holes flowing into the residue and red bars correspond to holes flowing out. Error bars correspond to one standard deviation.

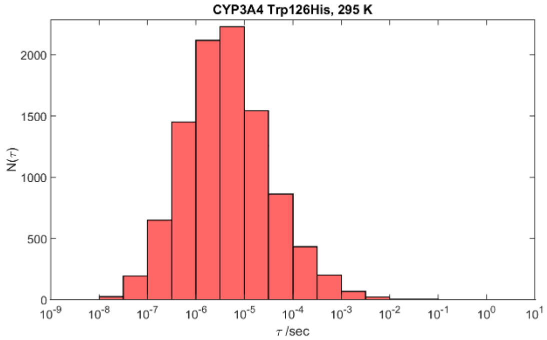

Figure S37. Distributions of calculated CI lifetimes (τ) for CYP3A4 Trp126His mutant at 295 K. Mean value is 4.5 μs.

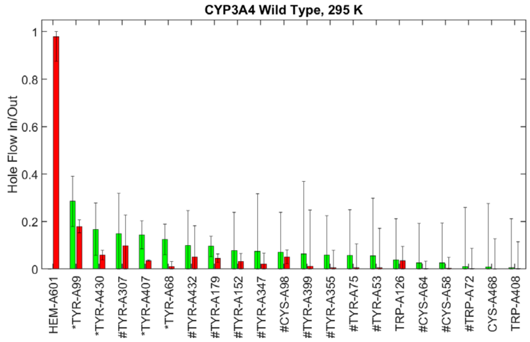

Figure S38. Calculated hole flow for redox active residues in CYP3A4 Trp126His mutant. Green bars correspond to holes flowing into the residue and red bars correspond to holes flowing out. Error bars correspond to one standard deviation.

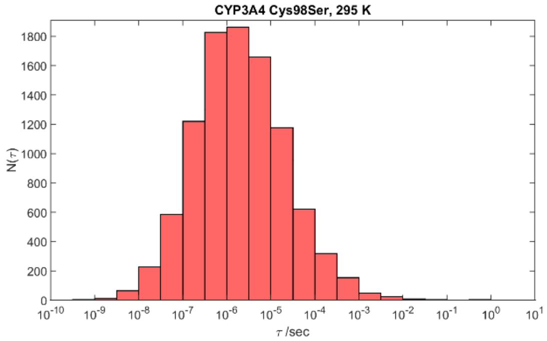

Figure S39. Distributions of calculated CI lifetimes (τ) for CYP3A4 Cys98Ser mutant at 295 K. Mean value is 2.1 μs.

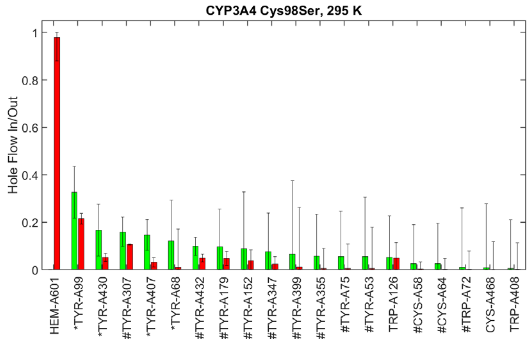

Figure S40. Calculated hole flow for redox active residues in CYP3A4 Cys98Ser mutant. Green bars correspond to holes flowing into the residue and red bars correspond to holes flowing out. Error bars correspond to one standard deviation.

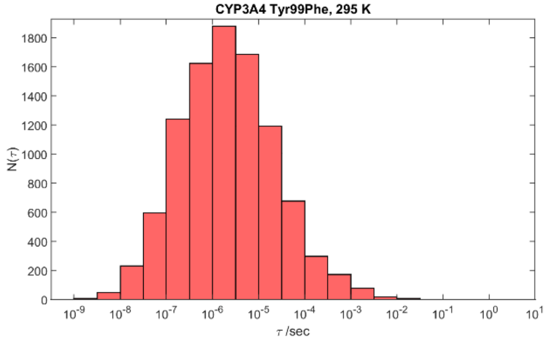

Figure S41. Distributions of calculated CI lifetimes (τ) for CYP3A4 Tyr99Phe mutant at 295 K. Mean value is 2.2 μs.

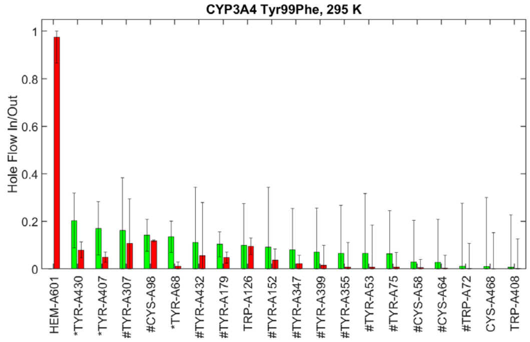

Figure S42. Calculated hole flow for redox active residues in CYP3A4 Tyr99Phe mutant. Green bars correspond to holes flowing into the residue and red bars correspond to holes flowing out. Error bars correspond to one standard deviation.

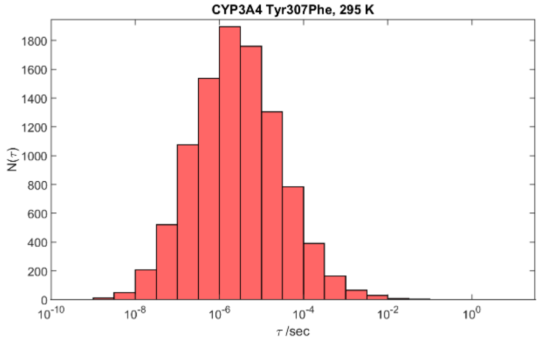

Figure S43. Distributions of calculated CI lifetimes (τ) for CYP3A4 Tyr307Phe mutant at 295 K. Mean value is 2.7 μs.

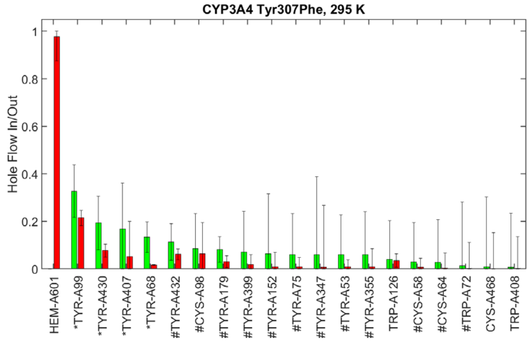

Figure S44. Calculated hole flow for redox active residues in CYP3A4 Tyr307Phe mutant. Green bars correspond to holes flowing into the residue and red bars correspond to holes flowing out. Error bars correspond to one standard deviation.

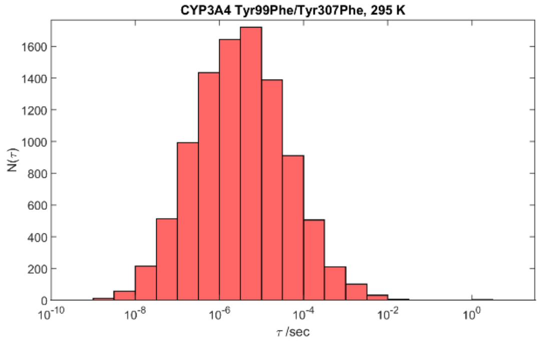

Figure S45. Distributions of calculated CI lifetimes (τ) for CYP3A4 Tyr99Phe/Tyr307Phe double mutant at 295 K. Mean value is 3.2 μs.

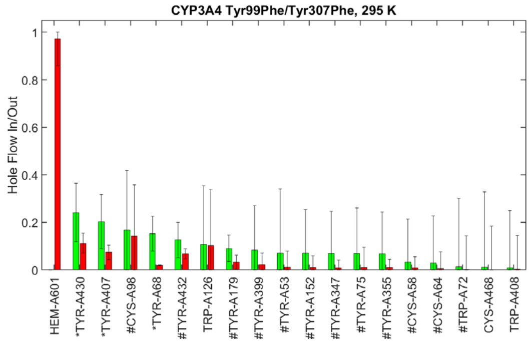

Figure S46. Calculated hole flow for redox active residues in CYP3A4 Tyr99Phe/Tyr307Phe double mutant. Green bars correspond to holes flowing into the residue and red bars correspond to holes flowing out. Error bars correspond to one standard deviation.

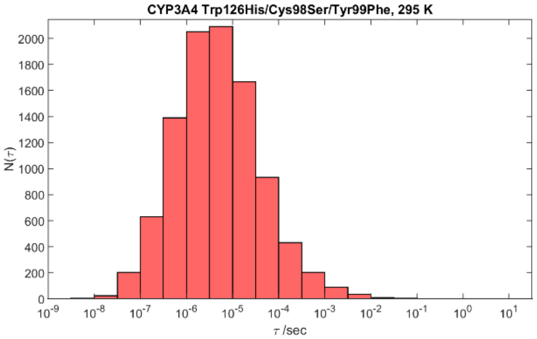

Figure S47. Distributions of calculated CI lifetimes (τ) for CYP3A4 Trp126His/Cys98Ser/Tyr99Phe triple mutant at 295 K. Mean value is 4.9 μs.

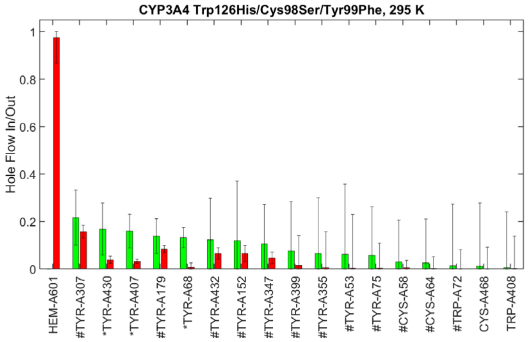

Figure S48. Calculated hole flow for redox active residues in CYP3A4 Trp126His/Cys98Ser/Tyr99Phe triple mutant. Green bars correspond to holes flowing into the residue and red bars correspond to holes flowing out. Error bars correspond to one standard deviation.
